# Insights from individuals successfully recovered from cannabis use disorder: natural versus treatment-assisted recoveries and abstinent versus moderation outcomes

**DOI:** 10.1186/s13722-018-0118-0

**Published:** 2018-07-30

**Authors:** David C. Hodgins, Jonathan N. Stea

**Affiliations:** Department of Psychology, University of Calgary, 2500 University Drive NW, Calgary, AB T2N 1N4 Canada

**Keywords:** Cannabis, Marijuana, Cannabis use disorder, Natural recovery, Treatment, Recovery pathways

## Abstract

**Background:**

Increasing understanding of the pathways and processes of recovery from cannabis use disorder may help in designing effective and attractive interventions to promote recovery. We report insights from individuals who had successfully recovered from cannabis use disorder with a variety of pathways. Recovered individuals describe their perceptions of why they developed the problem, why they were successful in recovering, and the advice they would offer to individuals with similar problems.

**Methods:**

Media announcements were used to recruit 119 volunteers who met lifetime but not past year criteria for cannabis use disorder. Participants were asked open-ended questions which were content analyzed and compared between individuals who whose recoveries were treatment-assisted (45%) versus natural (55%) and between individuals who were abstinent (57%) versus those who continued non-problematic consumption (43%).

**Results:**

Participants most frequently described their problems as having developed due to the use of cannabis to *cope*, because of *environmental and social influences*, and *enjoyment of the positive effects*. Success in recovery was attributed to *focusing on reasons for change*, *goal commitment to change*, and *conquering denial/self*-*deception*. Treatment-assisted participants were more likely to perceive that they overcame their cannabis problem due to *treatment/self*-*help* and *conquering underlying issues*, whereas naturally recovered participants were more likely to describe *focusing on reasons for change*, *will power*, and *lost enjoyment/lifestyle change*. Treatment-assisted participants were more likely to recommend *seeking help/social support* and naturally recovered participants were more likely to endorse reflecting *on reasons for change*, *engaging in hobbies/distracting activities*, and *stimulus control/avoidance/change social environment*. The majority recommended professional treatment (79.1%) and self-help materials (76.9%), and a little over half (53.2%) would also recommend natural recovery.

**Conclusions:**

These insights from people with lived experience further support previous research that treatment-assisted and natural recoveries are for the most part similar with respect to the recovery process. However, participants, whether or not they had had treatment involvement, recommended the use of treatment and self-help materials to sharpen their focus on the reasons to change and to enhance their commitment to change. At the same time, they saw value in the efforts of individuals to recovery without help.

**Electronic supplementary material:**

The online version of this article (10.1186/s13722-018-0118-0) contains supplementary material, which is available to authorized users.

## Background

Rates of cannabis use and cannabis use disorder (CUD) are rising in North America, and CUD is a common presenting issue in addiction treatment [20, 32]. However, treatment-seeking rates are low relative to the numbers of people with the disorder [17] and rates of natural recovery are significant [3, 8]. Increasing our understanding of the pathways and processes of recovery may help in designing effective and attractive interventions to promote recovery.

A growing body of literature supports the efficacy of a variety of interventions for CUD [14]. However, only a few empirical studies of the recovery process from CUDs have been reported. Cunningham et al. [9] interviewed former frequent cannabis users about their reasons for stopping. The most common explanations included emotional maturation and taking on new roles and responsibilities. Boyd et al. [4] interviewed cannabis users who had quit using at least once in the past about change strategies. Participants described changing their environment to be the most helpful strategy and help from professionals as the least helpful strategy. Ellingstad interviewed a more defined group of former daily cannabis users who had quit for at least 1 year without the assistance of treatment [11]. The major reasons described for resolving their problem were increasingly viewing cannabis as negative, experiencing more negative effects, and change being related to a broader lifestyle shift. Strategies cited as helpful in quitting included involvement in activities unrelated to cannabis use, avoidance of triggers to use, and lifestyle changes. Finally, in the only prospective study, Swift et al. [30] followed a group of 200 long-term cannabis users for a 1-year period during which time nearly two-thirds attempted to reduce or stop using. The top reasons for these change attempts were physical or psychological health effects, boredom with using, concerns they were using too much, lack of money, and lifestyle circumstances.

One limitation of the existing research is that samples generally include individuals with relatively brief periods of reduction or cessation of use, and therefore, the results may not be relevant for understanding the longer-term recovery process, which typically occurs after multiple change attempts. The only study that focused on stable recoveries, specifically, is the Ellingstad study [11] of abstinent individuals who had naturally recovered (i.e., did not attend treatment). We extended this research by comparing the experiences of individuals who had recovered with and without the assistance of treatment, either though abstinence from any use or moderation of use [29]. The treatment-assisted and abstinence groups exhibited higher levels of lifetime severity of cannabis problems than the natural and moderation groups. This association between severity of problem and recovery pathways has been found for other addictive behaviours including alcohol, heroin, and gambling [3]. Interestingly, however, despite the different pathways to recovery, the motivators and processes of recovery were mostly similar for the different groups. Regardless of whether they sought treatment or not, or whether they become abstinent or moderated their use, recovered individuals most frequently described their motivations for tackling recovery as a conscious choice that was related to their cannabis use becoming incompatible with their self-image and lifestyle, and related to their use causing negative psychological concerns. They described relying most frequently on cognitive strategies such as thinking about the negative consequences of their use or the benefits of less use as well as a variety of behavioural strategies (decreasing time spent with other users, avoidance of cues to use, involvement in incompatible activities).

These descriptions of pathways and processes of recovery are potentially helpful in the development of treatment approaches and treatment system policies. For example, the association between severity and treatment pathway is consistent with the tenets of stepped care models that offer increasingly intense treatment for increasingly severe problems. However, given that the treatment processes appear similar in the different pathways, it is important to delve deeper into individual experiences for a more complete understanding of the recovery process. In the present report we describe further insights from individuals who have successfully recovered from cannabis use disorder, contrasting treatment-assisted and natural pathways, and abstinent and moderation outcomes. Comparing these groups potentially provides the broad perspective necessary to conceptualize treatment systems. Specifically, we summarize individual perceptions of why they developed the problem, why they were successful in recovering, and the advice they would offer to individuals with similar problems. The perspective of people with lived experience is increasingly recognized as central to planning interventions that are both effective and attractive [24].

## Methods

### Participants

Media announcements were used to recruit 119 volunteers for an in-person interview who met lifetime but not past year criteria for cannabis use disorder [2], according to the Composite International Diagnostic Interview (CIDI) [21]. Recruitment ads asked for individuals who were willing to share information about their recovery, which might be helpful in designing future treatment programs for other people. Other inclusion criteria were age 18 years and older, ability to read and write English, and willingness to refrain from any alcohol or illicit drug use for at least 8 h prior to the interview. In addition, participants were asked to provide, if possible, the name of a family member or friend who would be able to corroborate their cannabis use history.

The sample was 30% female and 70% male with a mean age of 37.3 years. The mean age of first use of cannabis was 14.7 years (SD = 3.0) and the mean age of onset of CUD was 20.0 years (SD = 6.5). The median length of recovery was 5 years. A history of treatment was reported by 53 individuals (45%). Sixty-eight (57%) were categorized as abstinent (no cannabis use for at least 12 months) and 51 (43%) reported some non-problematic use. Individuals reporting use most frequently reported using once or twice in the past 12 months (59%) or monthly (20%), although 6% reported weekly use and 16% almost daily use. Other demographic details are provided in Stea et al. [29] and Additional file 1: Table S1.

### Interview

As the final section of a longer in-person interview that queried in detail cannabis use history, mental health comorbidity, and recovery efforts, and strategies, participants were asked a series of open-ended questions about the recovery process. Responses were probed for clarity and were recorded in writing by the interviewer. A number of areas were queried. First participants were asked: “What advice would you give to help another person with a marijuana problem”? Next they were asked whether they would recommend another person to reduce use to non-problematic levels or to quit completely (and why), and whether they would recommend that another person seek professional help or self-help materials or overcome the problem on their own without professional assistance. Participants were asked to describe their understanding of why they personally developed a marijuana problem, and finally, they were asked to describe their understanding of why they were able to overcome their problem. Participants could provide multiple responses to each question.

### Data analysis

Responses were content analysed [5] with the aid of NVivo software [25]. An inductive approach was used as no a priori hypotheses were proposed. One individual derived categories reflecting responses to each of the above questions (JS), and then an independent rater used the category labels and a brief one sentence category description to code each response. Inter-rater reliability was assessed using percentage agreement and Kappa coefficients. Kappa greater than .80 was set as the minimum level of agreement a priori. Disagreements were resolved through discussion to provide a final categorization of each response. Frequency counts were calculated for the total sample, and for abstinence versus moderation subgroups (recovery use status), and the treatment-assisted versus natural recovery subgroups (recovery pathway), which were compared using Chi squared tests, with Bonferroni correction to the alpha level according to the number of categories. In the case where a significant result was obtained on a particular dependent variable for both recovery use status and recovery pathway, follow-up tests were conducted that controlled for the effects of each respective factor in order to elucidate the relationship with the dependent variable.

## Results

### Perceived etiology

Ten categories of perceived personal etiology were uncovered and interrater reliability was excellent (percent agreement = 89.8, Kappa = .88). Participants identified a mean of 1.6 (SD = 0.7) categories and group comparisons revealed no differences between the abstinence- (*M* = 1.7, *SD* = 0.7) and moderation (*M* = 1.6, *SD* = 0.8) recovery use status groups, *F* (1, 115) = 0.1, *ns*, or between the treatment-assisted (*M* = 1.7, *SD* = 0.8) and natural recovery (*M* = 1.6, *SD* = 0.7) pathways groups, *F* (1, 115) = 0.4, *ns*. Table 1 displays the percentage of participants that endorsed each category (Additional file 1: Table S2 provides category descriptions). The top three categories reported were: *used cannabis to cope* (43.7%)*, environment/social influence* (41.2%), and *enjoyment/boredom/positive perceptions of cannabis* (23.5%). The category of *used cannabis to cope* reflected participant responses wherein cannabis was used to escape, self-medicate, and avoid emotional problems. The category of *environment/social influence* reflected participant responses wherein peer pressure and being around cannabis users was thought to contribute to the development of the cannabis problem. The category of *enjoyment/boredom/positive perceptions of cannabis* included enjoyment of the high, boredom, and positive perceptions of cannabis being helpful or fun in some way (e.g., mind enhancing/expanding, creating a philosophical and/or silly environment) was thought to contribute to the development of the cannabis problem.

**Table 1:** Percentage of participants that endorsed perceived etiology categories for the total sample and group comparisons

Category (%)	Total sample (*N* = 119)	Recovery use status			Recovery pathway		
		AB (*n* = 68)	MOD (*n* = 51)	χ^2^	TAR (*n* = 53)	NR (*n* = 66)	χ^2^
Used cannabis to cope	43.7	47.1	39.2	0.7	54.7	34.8	4.7*
Environment/social influence	41.2	38.2	45.1	0.6	39.6	42.4	0.1
Enjoyment/boredom/positive perceptions of cannabis	23.5	20.6	27.5	0.8	13.2	31.8	5.7*
Addictive personality	14.3	11.8	17.6	0.8	17.0	12.1	0.6
Genetics/predisposition	10.1	14.7	3.9	3.7	20.8	1.5	12.0***
Habit/dependence/addiction	9.2	11.8	5.9	*ns* ^i^	7.5	10.6	*ns* ^i^
Loss of control	7.6	8.8	5.9	*ns* ^i^	9.4	6.1	*ns* ^i^
Cannabis per se causes the addiction	6.7	5.9	7.8	*ns* ^i^	1.9	10.6	*ns* ^i^
No problem actually existed	4.2	5.9	2.0	*ns* ^i^	0.0	7.6	*ns* ^i^
Denial/self-deception/ignorance/choice	3.4	1.5	5.9	*ns* ^i^	3.8	3.0	*ns* ^i^

Chi square values represent Pearson Chi square values. Absolute Chi square values are reported. *AB* abstinence; *MOD* moderation; *NR* natural recovery; *TAR* treatment-assisted recovery

^i^Fisher’s exact test was used instead of Pearson Chi square because expected cell counts were less than 5

*p < .05; **p < .01; ***p < .001

While no differences emerged between the recovery use status, three differences emerged between the recovery pathway. Specifically, the treatment-assisted group was more likely to endorse the categories of *used cannabis to cope* (54.7 vs. 34.8%) and *genetics/predisposition* (20.8 vs. 1.5%), whereas the natural recovery group was more likely to endorse the category of *enjoyment/boredom/positive perceptions of cannabis* (31.8 vs. 13.2%). With a Bonferroni correction for 10 comparisons (α = .005), only the difference between the treatment-assisted group and the natural recovery group on the *genetics/predisposition* category remained statistically significant.

### Perceived causes of recovery success

Twelve categories of participants’ perceived causes of their recovery success showed excellent inter-rater reliability (percentage agreement = 83.3%, Kappa = .81, see Additional file 1: Table S3 for category descriptions). Participants identified a mean of 1.5 (*SD* = 0.8) categories. Group comparisons on the mean number of categories revealed no differences between the abstinence- (*M* = 1.5, *SD* = 0.7) and moderation (*M* = 1.6, *SD* = 0.8) recovery use status groups, *F* (1, 115) = 0.1, *ns*, or between the treatment-assisted (*M* = 1.5, *SD* = 0.7) and natural recovery (*M* = 1.6, *SD* = 0.8) pathways, *F* (1, 115) = 0.2, *ns*. As can be seen in Table 2, the top three major categories reported by the total sample were: *focused on reasons for change* (36.1%)*, goal commitment to change* (31.9%), and *conquered denial/self*-*deception* (25.2%). The category of *focused on reasons for change* reflected participant responses wherein recovery success was attributed to thinking about reasons for change and goal pursuit. The category of *goal commitment to change* reflected participant responses wherein recovery success was attributed to having strong motivation and commitment to change. The category of *conquered denial/self*-*deception* reflected participant responses wherein recovery success was attributed to a found sense of self-awareness or realization that cannabis was a problem.

**Table 2:** Percentage of participants that endorsed perceived causes of recovery success categories for the total sample and group comparisons

Category (%)	Total sample (*N* = 119)	Recovery use status			Recovery pathway		
		AB (*n* = 68)	MOD (*n* = 51)	χ^2^	TAR (*n* = 53)	NR (*n* = 66)	χ^2^
Focused on reasons for change	36.1	35.3	37.3	0.0	22.6	47.0	7.5**
Goal commitment to change	31.9	30.9	33.3	0.1	35.8	28.8	0.7
Conquered denial/self-deception	18.5	14.7	23.5	1.5	18.9	18.2	0.0
Treatment/self-help	13.4	16.2	9.8	1.0	24.5	4.5	10.1***
Religious/spiritual guidance	12.6	14.7	9.8	0.6	13.2	12.1	0.0
Will power	10.1	13.2	5.9	1.7	3.8	15.2	4.2*
Lost enjoyment/lifestyle change	8.4	5.9	11.8	*ns* ^i^	1.9	13.6	s*^i^
Social support	8.4	7.4	9.8	*ns* ^i^	11.3	6.1	*ns* ^i^
Stimulus control/avoidance/changed social environment	5.9	4.4	7.8	*ns* ^i^	5.7	6.1	*ns* ^i^
Conquered underlying issues	5.0	7.4	2.0	*ns* ^i^	11.3	0.0	s**^i^
Luck/lack of cravings or withdrawal	3.4	1.5	5.9	*ns* ^i^	3.8	3.0	*ns* ^i^
Helping others	1.7	2.9	0.0	*ns* ^i^	0.0	3.0	*ns* ^i^

Chi square values represent Pearson Chi square values. Absolute Chi square values are reported. *AB* abstinence; *MOD* moderation; *NR* natural recovery; *TAR* treatment-assisted recovery

^i^Fisher’s exact test was used instead of Pearson Chi square because expected cell counts were less than 5

*p < .05; **p < .01; ***p < .001

While no differences emerged between the recovery use status groups, five differences emerged between the recovery pathways groups. Specifically, the treatment-assisted group was more likely to endorse the categories of *treatment/self*-*help* (24.5 vs. 4.5%) and *conquered underlying issues* (11.3 vs. 0.0%), whereas the natural recovery group was more likely to endorse the categories of *focused on reasons for change* (47.0 vs. 22.6%), *will power* (15.2 vs. 3.8%), and *lost enjoyment/lifestyle change* (13.6 vs. 1.9%). With a Bonferroni correction for 12 comparisons (α = .004), only the difference between the treatment-assisted group and the natural recovery group on the *treatment/self*-*help* category remained statistically significant.

### Recovery advice

#### Strategies

Content analysis was used to derive thirteen categories of hypothetical advice provided by participants to help another person with a cannabis problem. Excellent inter-rater reliability was obtained (percentage agreement = 91.0%, Kappa = .90, see Additional file 1: Table S4 for category descriptions). Participants identified a mean of 1.8 (*SD* = 1.7) pieces of advice, with no differences between the abstinence- (*M* = 1.9, *SD* = 0.9) and moderation (*M* = 1.7, *SD* = 0.9) use status groups, *F* (1, 115) = 2.3, *ns*, or between the treatment-assisted (*M* = 1.7, *SD* = 0.9) and natural recovery (*M* = 1.9, *SD* = 0.9) groups, *F* (1, 115) = 0.8, *ns.* As can be seen in Table 3, the top three major advice categories reported by the total sample were: *seek help/social support* (37.8%)*, reflect on reasons for change* (26.1%), and *engage in hobbies/distracting activities* (25.2%). The category of *seek help/social support* reflected advice to seek a variety of forms of help, including from friends, family, and/or formal or professional help. The category of *reflect on reasons for change* included advice to think about the negative consequences of cannabis use, the reasons to change, and how life can be better without cannabis. The category of *engage in hobbies/distracting activities* reflected advice to occupy oneself via the pursuit of goals, hobbies, and other activities.

**Table 3:** Percentage of participants that endorsed advice for the total sample and group comparisons

Category (%)	Total sample (*N* = 119)	Recovery use status			Recovery pathway		
		AB (*n* = 68)	MOD (*n* = 51)	χ^2^	TAR (*n* = 53)	NR (*n* = 66)	χ^2^
Seek help/social support	37.8	47.1	25.5	5.8*	64.2	16.7	28.2***
Reflect on reasons for change	26.1	22.1	31.4	1.3	17.0	33.3	4.1*
Engage in hobbies/distracting activities	25.2	23.5	27.5	0.2	15.1	33.3	5.2*
Stimulus control/avoidance/change social environment	21.8	19.1	25.5	0.7	13.2	28.8	4.2*
Think positively	14.3	14.7	13.7	0.0	9.4	18.2	1.8
Face denial/self-deception	13.4	16.2	9.8	1.0	13.2	13.6	0.0
Change is a personal decision	11.8	13.2	9.8	0.3	7.5	15.2	*ns* ^i^
Find underlying issue/motive for use	9.2	8.8	9.8	*ns* ^i^	13.2	6.1	*ns* ^i^
Quit	7.6	10.3	3.9	*ns* ^i^	5.7	9.1	*ns* ^i^
Research cannabis/addiction	5.0	4.4	5.9	*ns* ^i^	5.7	4.5	*ns* ^i^
Seek spiritual/religious guidance	5.0	7.4	2.0	*ns* ^i^	3.8	6.1	*ns* ^i^
Moderate use	2.5	4.4	0.0	*ns* ^i^	3.8	1.5	*ns* ^i^
Not sure/no answer	2.5	1.5	3.9	*ns* ^i^	5.7	0.0	*ns* ^i^

Chi square values represent Pearson Chi square values. Absolute Chi square values are reported. *AB* abstinence; *MOD* moderation; *NR* natural recovery; *TAR* treatment-assisted recovery

^i^Fisher’s exact test was used instead of Pearson Chi square because expected cell counts were less than 5

*p < .05; **p < .01; ***p < .001

Several differences emerged among the groups. With respect to recovery status type, there was only one difference; the abstinence-oriented group was more likely to endorse the category of *seek help/social support* compared to the moderation-oriented group (47.1 vs. 25.1%); however, when recovery pathway was controlled for, there was no longer a significant relationship [treatment-assisted participants, χ^2^ (1) = 3.2, *ns*; naturally recovered participants, χ^2^ (1) = 0.2, *ns*]. With respect to recovery pathway, the treatment-assisted group was more likely to endorse the category of *seek help/social support* (64.2 vs. 16.7%)—this relationship remained significant after controlling for recovery use status [treatment-assisted participants, 72.2 vs. 18.8%, χ^2^ (1) = 19.4, *p* < .001; naturally recovered participants, 47.1 vs. 14.7%, Fisher’s exact test, *sig.*]—whereas the natural recovery group was more likely to endorse the categories of *reflect on reasons for change* (33.3 vs. 17.0%), *engage in hobbies/distracting activities* (33.3 vs. 15.1%), and *stimulus control/avoidance/change social environment* (28.8 vs. 13.2%). With a Bonferroni correction for 13 comparisons (α = .004), only the difference between the treatment-assisted group and the natural recovery group on the *seek help/social support* category remained statistically significant.

#### Pathways

Participants were also asked to provide a number of recommendations about different recovery pathways, the results of which are displayed in Table 4. The majority of the total sample reported that they would recommend professional treatment (79.1%) and self-help materials (76.9%), and a little over half (53.2%) would recommend natural recovery (i.e., that a hypothetical person with a cannabis problem try to overcome the problem on their own without professional assistance). Interestingly, the moderation status group was significantly more likely to recommend natural recovery than the abstinent group (67.4 vs. 42.9%); however, when recovery pathway was controlled for, this relationship only remained significant among treatment-assisted participants (53.3 vs. 20.0%, Fisher’s exact test, *sig.*), not naturally recovered participants [χ^2^ (1) = 0.0, *ns*]. With respect to the recovery pathway group, the treatment-assisted group was significantly more likely to recommend self-materials (86.8 vs. 68.8%), whereas the natural recovery group was significantly more likely to recommend natural recovery (72.9 vs. 30.0%); however, when recovery use status was controlled for, this latter relationship only remained significant among abstinence-oriented participants [71.4 vs. 20.0%, χ^2^ (1) = 16.8, *p* < .001], not moderation-oriented participants (Fisher’s exact test, *ns*).

**Table 4:** Participant recommendations for the total sample and group comparisons

Variable	Total sample (*N* = 119)	Recovery use status			Recovery pathway		
		AB (*n* = 68)	MOD (*n* = 51)	χ^2^	TAR (*n* = 53)	NR (*n* = 66)	χ^2^
Recommend professional treatment for cannabis problem (% yes)	79.1^a^	79.1^b^	79.2^c^	0.0	86.5^d^	73.0^e^	3.1
Recommend self-help materials for cannabis problem (% yes)	76.9^f^	79.4	73.5^g^	0.6	86.8	68.8^h^	5.3*
Recommend natural recovery for cannabis problem (% yes)	53.2^j^	42.9^e^	67.4^k^	6.4*	30.0^l^	72.9^m^	20.0***
Recommend AB versus MOD (%)							
Quit	48.7	58.8	35.3	6.5*	62.3	37.9	7.0**
Depends on the person	24.4	19.1	31.4	2.4	17.0	30.3	2.8
Reduce/cut-back	19.3	13.2	27.5	3.8*	15.1	22.7	1.1
Neither or both	7.6	8.8	5.9	*ns* ^i^	5.7	9.1	*ns* ^i^

Chi square values represent Pearson Chi square values. Absolute Chi square values are reported. *AB* abstinence; *MOD* moderation; *NR* natural recovery; *TAR* treatment-assisted recovery

^i^Fisher’s exact test was used instead of Pearson Chi square because expected cell counts were less than 5

^a^*n* = 115

^b^*n* = 67

^c^*n* = 48

^d^*n* = 52

^e^*n* = 63

^f^*n* = 117

^g^*n* = 49

^h^*n* = 64

^j^*n* = 109

^k^*n* = 46

^l^*n* = 50

^m^*n* = 59

*p < .05; **p < .01; ***p < .001

When asked whether a person should reduce/cut-back or quit their cannabis use completely, the most popular response was to *quit* (48.7%), followed by *depends on the person* (24.4%), *reduce/cut back* (19.3%), and *neither or both* (7.6%). The abstinence-oriented group was significantly more likely to recommend that people quit (58.8 vs. 35.3%); however, when recovery pathway was controlled for, this relationship no longer remained significant [treatment-assisted participants, χ^2^ (1) = 2.5, *ns*; naturally recovered participants, χ^2^ (1) = 2.1, *ns*]. Moreover, the moderation status group was significantly more likely to recommend that people reduce/cut-back (27.5 vs. 13.2%). With respect to recovery pathway, the treatment-assisted group was significantly more likely to recommend that people quit compared to the natural recovery group (62.3 vs. 37.9%); however, when recovery status was controlled for, this relationship no longer remained significant [abstinence-oriented participants, χ^2^ (1) = 3.6, *ns*; moderation-oriented participants, χ^2^ (1) = 1.5, *ns*].

## Discussion

Understanding the perceptions of people with lived experience about the development and resolution of a CUD is instructive in conceptualizing and promoting the process of recovery. Individuals with stable recoveries are one important informant group, but it is important that different experiences be represented, including different pathways and both abstinent and moderation outcomes. In this study, recovered individuals described their problems as having developed for multiple reasons, but in particular due to the use of cannabis to cope, because of environmental and social influences, and enjoyment of the positive effects. These perceptions align with research into the etiology of CUD insofar as coping motives for cannabis use have been found to be associated with the development of cannabis use disorders [6, 13]; environmental and social influences such as low parental monitoring, high peer group deviance, and cannabis availability have been found to predict cannabis initiation [15]; and perceived risk of harm has been found to be inversely associated with frequency of cannabis use [10, 12, 19]. Interestingly, we previously reported the responses of this sample to the Marijuana Motives Measure (MMM, [27]), which indicated that participants most often used cannabis for enhancement motives during their lifetime, followed by social, coping, expansion, and conformity motives [29]. This suggests that while participants believed that coping motives were the strongest contributor to the development of their cannabis problem, they more often used cannabis for other purposes (i.e., enhancement and social reasons), which highlights the pervasiveness of cannabis use during their lifetime.

Treatment-assisted participants were more likely to endorse *genetics/predisposition* compared to naturally recovered participants, whereas the latter group was more likely to endorse the reason of *enjoyment/boredom/positive perceptions of cannabis*. The fact that treatment-assisted participants more readily identified *genetics/predisposition* as an etiological category might reflect that they were taught this addiction model in treatment, but it is also possible that their CUDs were relatively and genuinely more influenced by genetic factors in light of their more frequent reports of family addiction problems [28]. There is indeed solid evidence that cannabis use and cannabis use disorders have heritable components [1, 33].

Participants were also asked to explain their understanding of why they were able to overcome their cannabis problem. The content analysis revealed that the top cited reasons were *focusing on reasons for change*, *goal commitment to change*, and *conquering denial/self*-*deception*. While there were no differences with respect to the recovery use status, treatment-assisted participants were more likely to perceive that they overcame their cannabis problem due to reasons of *treatment/self*-*help* and *conquered underlying issues*, whereas naturally recovered participants were more likely to cite the categories of *focused on reasons for change*, *will power*, and *lost enjoyment/lifestyle change*. These results suggest that participants attributed their recovery success to cognitive and motivational factors, which is consistent with our previous analyses demonstrating that cognitive strategies were the most helpful actions taken and maintenance factors involved in recovery [29]. These results support CBT-MET approaches to the treatment of CUDs [7, 14], and might suggest that an increased cognitive and motivational focus might be one way to improve psychosocial treatments [29].

Finally, participants were asked to provide advice and recommendations to help another hypothetical person with a cannabis problem. The top cited advice categories were to *seek help/social support*, *reflect on reasons for change*, and *engage in hobbies/distracting activities*. Interestingly, no differences emerged between the abstinence- and moderation-oriented participants. Whereas treatment-assisted participants were more likely to endorse the category of *seek help/social support* naturally recovered participants were more likely to endorse the categories of *reflect on reasons for change*, *engage in hobbies/distracting activities*, and *stimulus control/avoidance/change social environment*. These results differ from the advice provided by a sample of recovered gamblers participating in a treatment outcome study [31], whereby it was mostly indicated that there was nothing that could be done to aid in the decision to cease or reduce gambling, with a smaller proportion suggesting awareness-raising strategies (e.g., pointing out the negative consequences of problem gambling and arousing cognitive dissonance).

The majority of participants reported that they would recommend treatment and self-help materials to a hypothetical person with a cannabis problem. However, treatment-assisted participants were more likely to recommend self-help materials compared to naturally recovered participants, whereas the latter group was more likely to recommend natural recovery (i.e., try to resolve the cannabis problem without professional assistance), albeit only among those who were abstinent. Interestingly, moderation status participants who were treatment-assisted were more likely to recommend natural recovery than other participants, which might reflect a perceived lack of fit between their goals and the goals of the treatment program they attended. It is unclear to what extent these recommendations might be influenced in the future if moderation-focused treatments for cannabis use disorders become widely available. Additionally, the majority of participants reported that they would recommend abstinence to a hypothetical person with a cannabis problem, and not surprisingly, moderation status participants were more likely to recommend moderation compared to abstinent status participants.

These findings are limited in that the results are based on self-reports of volunteer participants. Use of open-ended questions with probing by interviews, and qualitative analysis is helpful given how little is known about the recovery process. However, it is impossible to know how representative these individuals are of the general recovery population, and how able individuals are at identifying recovery influences. In addition, although the cannabis use histories were corroborated by family and friend interviews [18], the findings are based upon participant retrospective attributions of events occurring a number of years ago. Assessing similar issues earlier in recovery may elicit somewhat different information.

## Conclusions

It is timely and important that we attend to how to best promote recovery from CUD. With the recent trend towards cannabis decriminalization and legalization, it remains unclear to what extent these legal changes might impact the incidence of cannabis use disorders. While the majority of individuals who use cannabis will not develop a problem, a substantial minority of individuals who will develop a problem deserve greater access to non-stigmatizing and improved treatment options. With a view towards informing evidence-based clinical practice, the present study explored the recovery process from cannabis use disorders in the context of multiple recovery pathways, the findings of which hold a number of implications for policy and practice. These insights from people with lived experience further support previous research that treatment-assisted and natural recoveries are for the most part similar with respect to the recovery process, which is consistent with Klingemann’s contention that there exists a shrinking gap between the natural recovery and treatment outcome literature [23]. The frequent attribution of the use of cannabis for coping with negative emotions reinforces the inclusion of emotional regulation training in treatment protocols and prevention efforts. The results for cannabis are also consistent with those for other substances, which supports the growing attention to transdiagnostic etiological and treatment models focusing on common elements [16, 22, 26].

However, participants, whether or not they had had treatment involvement, recommended the use of treatment and self-help materials to sharpen their focus on the reasons to change and to enhance their commitment to change. At the same time, they saw value in the efforts of individuals to recovery without help. As we have previously argued, these findings support the development of stepped care models that offer varying levels of support according to client wishes and needs [29]. Such models can include the provision of quality self-help materials by primary care providers and public health initiatives that promote self-directed efforts that are similar to treatment processes. The acknowledgement of moderation recovery outcomes within these models and self-help materials provides a more accurate and nuanced depiction of the recovery process, which might serve to de-stigmatize the non-isomorphic link between cannabis use and cannabis use disorder.

## Additional file

**Additional file 1.** Content analyses category descriptions.
