# Supplementary material for: Insights from individuals successfully recovered from cannabis use disorder: natural versus treatment-assisted recoveries and abstinent versus moderation outcomes
Source: Addict Sci Clin Pract. 2018 Jul 30;13:16. doi: 10.1186/s13722-018-0118-0 (PMC6065061; doi:10.1186/s13722-018-0118-0)
Supplement: Supplementary file 1 — Additional file 1. Content analyses category descriptions. [file 13722_2018_118_MOESM1_ESM.docx]

Table s1

*Demographics for the Total Sample and Group Comparisons*

|  |  | Recovery Use Status | | | Recovery Process | | |
| --- | --- | --- | --- | --- | --- | --- | --- |
| Variable | Total Sample (*N* = 119) | AB  (*n* = 68) | MOD  (*n* = 51) | *F*-test  / χ^2^ | TAR  (*n* = 53) | NR  (*n* = 66) | *F*-test / χ^2^ |
| Age in years, *M* (*SD*) | 37.4 (12.9) | 40.8 (13.5) | 32.8 (10.5) | 9.5^**i^ | 39.0 (13.0) | 36.1 (12.8) | 0.6^i^ |
| Gender (% male) | 70.0 | 67.6 | 72.5 | 0.3 | 64.2 | 74.2 | 1.4 |
| Marital status (%) |  |  |  | 4.0 |  |  | 3.7 |
| Single | 36.1 | 30.9 | 43.1 |  | 34.0 | 37.9 |  |
| Dating | 10.9 | 8.8 | 13.7 |  | 13.2 | 9.1 |  |
| Married | 20.2 | 25.0 | 13.7 |  | 20.8 | 19.7 |  |
| Common law | 16.8 | 17.6 | 15.7 |  | 11.3 | 21.2 |  |
| Separated/divorced/widowed | 16.0 | 17.6 | 13.7 |  | 20.8 | 12.1 |  |
| Education (%) |  |  |  | 2.6^ii^ |  |  | 2.9^ii^ |
| No degree/certificate/diploma | 13.4 | 14.7 | 11.8 |  | 17.0 | 10.6 |  |
| Secondary (high school) graduation  certificate/diploma | 30.3 | 33.8 | 25.5 |  | 30.2 | 30.3 |  |
| Trades certificate/diploma | 16.8 | 14.7 | 19.6 |  | 15.1 | 18.2 |  |
| Other non- university  certificate/diploma | 10.1 | 8.8 | 11.8 |  | 13.2 | 7.6 |  |
| University certificate/diploma below  bachelor level | 9.2 | 7.4 | 11.8 |  | 7.5 | 10.6 |  |
| Bachelor's degree | 12.6 | 11.8 | 13.7 |  | 11.3 | 13.6 |  |
| University certificate/diploma/degree  above bachelor level | 7.6 | 8.8 | 5.9 |  | 5.7 | 9.1 |  |
| Employment (%) |  |  |  | 3.1^ii^ |  |  | 3.0^ii^ |
| Employed full-time | 52.1 | 52.9 | 51.0 |  | 52.8 | 51.5 |  |
| Employed part-time | 10.1 | 13.2 | 5.9 |  | 5.7 | 13.6 |  |
| Unemployed | 10.9 | 8.8 | 13.7 |  | 13.2 | 9.1 |  |
| Student – employed | 12.6 | 13.2 | 11.8 |  | 11.3 | 13.6 |  |
| Student – unemployed | 8.4 | 7.4 | 9.8 |  | 9.4 | 7.6 |  |
| Other | 5.9 | 4.4 | 7.8 |  | 7.5 | 4.5 |  |
| Approx. net yearly income ($), *M* (*SD*) | 43168.32 (29415.01)^a^ | 45406.78 (32043.68)^b^ | 40023.81 (25307.61)^c^ | 0.3 | 46880.95 (31895.56)^c^ | 40525.42 (27487.68)^b^ | 0.9 |
| Self-identified ethnicity (%) |  |  |  | 0.2^ii^ |  |  | 0.1^ii^ |
| Caucasian | 79.8 | 79.4 | 80.4 |  | 79.2 | 80.3 |  |
| Aboriginal | 5.0 | 4.4 | 5.9 |  | 5.7 | 4.5 |  |
| Other | 15.1 | 16.2 | 13.7 |  | 15.1 | 15.2 |  |
|  |  |  |  |  |  |  |  |
|  |  |  |  |  |  |  |  |
|  |  |  |  |  |  |  |  |

*Note.* AB = abstinence; MOD = moderation; NR = natural recovery; TAR = treatment-assisted recovery.

^i^ Levene's test of equality of error variances was significant.

^ii^ Cells have an expected count less than 5.

^ii^ Scale: 1 = Not important at all; 2 = Not very important; 3 = Somewhat important; 4 = Very important.

^a^ *n* = 101. ^b^ *n* = 59. ^c^ *n* = 42.

^*^ *p* < .05. ^**^ *p* < .01. ^***^ *p* < .001.

Table S2

*Representative Content from the Perceived Etiology Categories*

| Category | Representative Content |
| --- | --- |
| Used cannabis to cope | - "I used it to medicate my emotions, to escape, not just to have a good time. I became dependent on it. I used it to self-medicate, avoid other problems...over 10 years, it manifests itself into an addiction."  - "Help deal with and mask low self-esteem, self-confidence."  - "I was filling a void that I couldn't get elsewhere."  - "I tried to escape and not deal with my emotions."  - "The stress relief, even though it was causing the stress. It's a catch 22." |
| Environment/  social influence | - "A lot of my peers were doing it, peer pressure. I was a teenager, peer pressure."  - "Where I grew up, it was a way of life, it was as normal as having a cigarette during break time at school."  - "Being around friends who use it."  - "Peer pressure...falls into not being comfortable in my own skin. I wanted to be accepted. I would do anything, get high to be cool."  - "Your environment, I was around it." |
| Enjoyment/  boredom/  positive perceptions of cannabis | - "I just really liked the feeling...that's about it. I really liked it but then it turned its back on me."  - "The perception that it is mind enhancing/expanding."  - "Enjoyed being high, excuse to be philosophical or silly."  - "Didn't have anything better to do."  - "I liked the feeling of being high, more so than the effects of alcohol." |
| Addictive personality | - "I think there is something to be said about addictive personality."  - "I used to have a really addictive personality, still kind of do."  - "I think I generally tend to have addictive personality.  - "Addictive personality, I was told at Fresh Start."  - "Highly addictive personality, anything I've done, I've done a lot." |
| Genetics/  predisposition | - "Might be genetic, hardwired. Some families have no addictions. It skips a generation in my family...maybe because each generation sees what it does."  - "It's a predisposition to addiction and mental illness. I'm just wired differently, it did something for me that I liked."  - "I think coming from parents that have addiction issues."  - "Vulnerable, genetically predisposed, family problems, susceptible. If not pot, it would have been something else."  - "Addiction is genetic, I had a predisposition to addiction." |
| Habit/  dependence/  addiction | - "Became a habit."  - "Regular use...body got used to...goes by in a blink, you never say, "I'll smoke every day of my life"."  - "Became a habit/addiction."  - "Years of use, years of relying on it."  - "Habit part of it...I was used to being stoned." |
| Loss of control | - "Feeling of not being able to stop."  - "Got out of control."  - "I constantly wanted to get back the same high."  - "I was obsessed with it."  - "It snowballed." |
| Cannabis per se causes the addiction | - "Marijuana has control of the biological/chemical imbalance."  - "It's addictive."  - "The amount used...change in potency, old versus new marijuana."  - "Maybe some bad dope, it could have been laced."  - "It will affect your mind, your decision making, then problems come out like a mushroom." |
| No problem actually existed | - "I never perceived it as a problem...meaning interfering with life, socially and professionally...I still don't have a problem."  - "I don't believe I had a problem, always felt in control of use, even at high levels of usage."  - "It never seemed like a problem. It was just a habit. It only became an issue when my girlfriend wanted to go into law enforcement."  - "I knew I could always manage what I was doing. I knew I was in change, and when I was going to stop, I would stop. I was quite conscious that I could have fun and then stop whenever I wanted."  - "I never considered it to be a problem. Others thought it was and tried to extend their feelings to change the way I see marijuana. They made me think it was a problem." |
| Denial/  self-deception/  ignorance/  choice | - "At the time, lack of feeling it was a problem, denial of any perceived problem."  - "Young and ignorant."  - "I chose it, decisions, that's all it comes down to."  - "I think that everyone has a problem with marijuana...everyone else is lying." |

Table S2

*Representative Content from the Perceived Causes of Recovery Success Categories*

| Category | Representative Content |
| --- | --- |
| Focused on reasons for change | - "Kept focused on the reasons I wanted to quit."  - "Having the confidence, maturity, and knowledge that it isn't doing me any good...and thinking about how it's not helping me achieve my goals, it's useless and pointless."  - "I had a reason, a goal in mind and I need to quit to accomplish that goal."  - "Determined to become successful in life, wealthy and prestigious. Wanting a more fulfilling life...using decreased self-esteem and mental/physical health."  - "Biggest reason is because I set goals and was determined to achieve them, slowing marijuana use was necessary to achieve them." |
| Goal commitment to change | - "Stuck to my guns, made sure I stuck to it and followed through."  - "Sheer motivation, I was that determined. Other people might not want to. They would self sabotage without even knowing it, this applies to all addictions."  - "Because I said I wanted to. I took the initiative and did it."  - "My decision and commitment to complete abstinence. Commitment/wanting to be clean, has to come 100% from you, not anyone else."  - "Very determined to overcome the problem." |
| Conquered denial/  self-deception | - "I had the ability to recognize I had a problem. I know lots of people who say they don't have a problem, but they do."  - "Once I saw what I had become, and didn't try to minimize the fact that it was just marijuana, I didn't want to be that anymore. It was a bigger problem than what it was worth physically and emotionally. I didn't like what I saw in the mirror, I had to change the picture."  - "The crux is the willingness to do things that are really uncomfortable, to be honest with myself and others."  - "I saw it as a lie, and as a coping mechanism that people use, like drinking."  - "I had self-awareness that marijuana was impeding the process of progressing as a human, the human I wanted to be." |
| Treatment/  self-help | - "I've been involved for 25 years in Narcotics Anonymous. Hearing people talk about it, I can't pin it down. I don't know. The only thing I can say is that the program of NA worked for me perfectly, I consider myself textbook. I even used to lie about marijuana in recovery because people didn't think of it as bad. But now, I'm fully on the other side, I always say it is my primary drug."  - "I had rehab tools, basically the whole package of rehab."  - "I attribute a lot of it to my treatment. I don't think Alcoholics and Narcotics Anonymous was enough, I think residential treatment was necessary to help me resolve my past pains and generate self-love. I like who I am today."  - "Education, access to a lot of materials...better able to make an informed decision."  - "Research, educating myself, reading materials, sharing." |
| Religious/  spiritual guidance | - "I think there is something bigger than us, a creator. I've always had a connection with it. I'm not religious, but I'm spiritual, and I believe in something else that is in control, and being able to access that has helped me to get passed this."  - "God had a purpose for me, marijuana was a distraction from God's plan."  - "I had Jesus."  - "Not very religious but felt profound chosen sense of need to quit. God spoke my name."  - "Religion played a part." |
| Will power | - "I'm strong willed."  - "Will power."  - "My drive and will power."  - "Strong willed."  - "Strong will power." |
| Lost enjoyment/  lifestyle change | - "I no longer like the high, it sucks. People who smoke like the high."  - "Change of circumstances, life became fulfilling without using...environmental changes, different friends, work environment."  - "It was a gradual to abrupt lifestyle change. When I quit, I quit."  - "Just didn't like it anymore."  - "Grew out of it, tired of the smell." |
| Social support | - "I had support of others."  - "Financially and emotionally supportive parents and family."  - "Positive people came into my life."  - "Very good personal supports."  - "Related to other people, realized that I wasn't alone, it was possible by seeing other people in the same situation." |
| Stimulus control/  avoidance/  changed social environment | - "I don't hang out with people at all anymore who use."  - "Avoiding people, situations."  - "Depends on the environment, took myself out of toxic environment."  - "Changed external environment."  - "Removing myself from the environment, not spending much time with friends." |
| Conquered underlying issues | - "I found the underlying issues that caused me to feel badly about myself, and I have healed from that. Therein, I filled that void of self-worth that was missing."  - "Because I found out why I was doing it and I sought treatment for that issue. And since I was seeking treatment for that issue, I could look at my marijuana use more objectively and see it wasn't helpful for anyone using it to cope with their issues."  - "Learned about underlying issues. Learned to cope with issues."  - "Dealt with core issues."  - "Was able to develop more mature emotional responses to my particular problems, instead of medicating with marijuana." |
| Luck/  lack of cravings or withdrawal | - "I'm one of the lucky ones. I would like to know why I overcame it as well."  - "Fortunately, withdrawal symptoms were minimal."  - "I just never had strong cravings or urges when trying to quit."  - "Different physiology, did not have addicted personality like others do." |
| Helping others | - "Counselling of other people with the same problem."  - "Being a positive influence for others, being a good example." |

Table S3

*Representative Content from the Advice Categories*

| Category | Representative Content |
| --- | --- |
| Seek help/  social support | - "Find some people that know about addictions, especially marijuana."  - "Seek outside help, specifically Narcotics Anonymous."  - "Open up, talk to someone."  - "Don't be afraid to seek help if you need it."  - "Nothing wrong with seeking professional help." |
| Reflect on reasons for change | - "Encourage them to find a reason to reduce their use (e.g., family, religion, etc.)."  - "Weigh out your pros and cons."  - "Weed is not helping your life, it's temporary entertainment that is really costly and bad for your health, and after a certain point, it will make you feel worse."  - "Life is actually better without marijuana."  - "Re-evaluate whether they can achieve their life goals at their usage." |
| Engage in hobbies/  distracting activities | - "Find a project or goal that they are inspired by or motivated by."  - "Get busy, find activities, something to do."  - "Set goals, short term and long term, make them realistic."  - "Get new hobbies, change of life style."  - "Change your lifestyle...exercising, school, job, better yourself." |
| Stimulus control/  avoidance/  change social environment | - "I would suggest to avoid situations and people they use with."  - "Get rid of your paraphernalia."  - "I would recommend moving to a different environment, different areas."  - "Change your friends, develop a good support network."  - "Don't hang out with people who use." |
| Think positively | - "If you stick to it, it can be overcome, be positive with it...it's like cigarette smoking, if you can quit smoking, you can quit smoking marijuana."  - "Use your will power, retrain your brain."  - "Think highly of yourself, you can do anything you want to do."  - "To do everything, you've got to believe in yourself that you have the right attitude...you need it, it's always a mind game."  - "Persistence conquers all things." |
| Face denial/self-deception | - "If someone recognizes the problem, that's half the battle"  - "Being honest about why you're using."  - "Be honest with themselves, no denial."  - "Admit you have a problem and want/need to change."  - "Don't lie to yourself, identify the level of addiction." |
| Change is a personal decision | - "Need to make a choice for yourself. It's your decision, just do it."  - "You have to want to quit."  - "People can't change unless they want to."  - "If you want to stop it's up to you, no one but you can make you stop."  - "You control your life, grow up and quit. It's all on you, quit if you want to quit." |
| Find underlying issue/motive for use | - "Marijuana use is not the underlying problem. What is the drug doing for you? Marijuana is a medication. The real question is what are you medicating for?"  - "First ask yourself, why do you like it? Are you hiding from something? Then, if you can come up with an answer, and it's because you're hiding, figure out what you're hiding from and why you feel that way."  - "Ask yourself why you are using it, you won't be able to fix it unless you know why you have the problem."  - "Person needs to find out why they use."  - "There is probably some deep seeded reason why...try to get to the bottom of it. Ask the question, why do you want to be high?" |
| Quit | - "You should quit."  - "It's all or none, can't occasionally use."  - "Don't do it once, you will be back to problem using."  - "Need to quit completely."  - "If you can quit, just quit." |
| Research cannabis/addiction | - "Educate yourself about marijuana."  - "Read books on addiction and cannabis; for example, From Chocolate to Morphine."  - "Education, and support and encourage them to educate themselves."  - "If they have a problem, I'd say read something...it's obviously a problem with themselves."  - "Do some research." |
| Seek spiritual/  religious guidance | - "There is a need for a spiritual life."  - "Pray to whatever."  - "Seek God."  - "Pick up a Bible."  - "Look to religion, spirituality." |
| Moderate use | - "How do you convince someone to quit when it's becoming legal? Do what works for you, don't distress yourself, cut back on weekends if you have to."  - "Don't smoke it, eat it if you have to."  - "Consider it a process and gradually decrease the usage while keeping in mind that it's a process and be willing to accept the sliding scales of the process." |
